# A Comparative Evaluation of Smear Layer Removal Using Apical Negative Pressure (EndoVac), Sonic Irrigation (EndoActivator) and Er:YAG laser -An *In vitro* SEM Study

**DOI:** 10.4317/jced.53881

**Published:** 2017-08-01

**Authors:** Sanghamitra Suman, Promila Verma, Aseem Prakash-Tikku, Rhythm Bains, Vijay Kumar-Shakya

**Affiliations:** 1 Post Graduate Student, Department of Conservative Dentistry & Endodontics, Faculty of Dental Sciences, King George's Medical University, Lucknow, Uttar Pradesh, India; 2 Professor, Department of Conservative Dentistry & Endodontics, Faculty of Dental Sciences, King George's Medical University, Lucknow, Uttar Pradesh, India; 3 Associate Professor, Department of Conservative Dentistry & Endodontics, Faculty of Dental Sciences, King George's Medical University, Lucknow, Uttar Pradesh, India; 4 Assistant Professor, Department of Conservative Dentistry & Endodontics, Faculty of Dental Sciences, King George's Medical University, Lucknow, Uttar Pradesh, India

## Abstract

**Background:**

This study aimed to compare the smear layer removing efficacy of the EndoActivator, EndoVac and Er:YAG laser in extracted mandibular premolars, at the apical, middle and coronal third of root canal, through scanning electron microscopy.

**Material and Methods:**

40 extracted mandibular premolars were decoronated to a standardized length of 12 mm. Specimens were shaped to ProTaper F4 size and irrigated with 5.25% sodium hypochlorite at 370C between instrumentation. Teeth were divided into four groups (n=10), one control (needle irrigation) and three experimental, according to the irrigant activation technique used i.e. sonic irrigation (EndoActivator), apical negative pressure (EndoVac) or laser (Er:YAG). The final irrigants used were 10ml,17% ethylenediaminetetraacetic acid (EDTA) and 10ml, 5.25% sodium hypochlorite. Root canals were then split longitudinally and observed under a scanning electron microscope. The presence of smear layer at the apical, middle and coronal third of root canal was evaluated. Scores were analyzed by Kruskal-Wallis and Mann-Whitney U tests. Intraexaminer and interexaminer reliability were determined by Kappa test.

**Results:**

The EndoVac system was significantly more effective in removing debris from the apical third than all other groups. EndoActivator performed better than laser at the apical third. All three experimental groups (EndoVac, EndoActivator, and laser) were better than needle irrigation at the middle and apical third. At the coronal third, no significant difference was seen between the four groups.

**Conclusions:**

None of the activation systems completely removes the smear layer from the dentine walls; nevertheless, EndoVac is significantly better in removing debris from the apical third of canal.

** Key words:**EndoVac, EndoActivator, Er:YAG laser, smear layer, scanning electron microscopy.

## Introduction

One of the prerequisites of a successful endodontic treatment is an efficient removal of smear layer from the dentinal walls. A complete debridement of the root canal is essential to achieve an effective disinfection and a three-dimensional obturation for a favorable long-term prognosis (1-3).

Traditional needle irrigation has been proved to be insufficient for a complete cleaning of the complex anatomy of root canal system (especially the lateral canals, isthmuses and the apical third), therefore endeavors are being made to develop new irrigants and irrigating devices to improve the root canal disinfection in everyday endodontic practice (4-6). Apical negative pressure (EndoVac), sonic activation (EndoActivator) and Er: YAG laser are three such promising techniques that claim to improve the irrigant’s effectiveness particularly at the apical third of canal. EndoActivator (EA) ((Dentsply, Tulsa Dental Specialties, Tulsa, OK), the sonically driven irrigant activation system, works on the principle of sonic activation of files (1-6 kHz) to produce hydrodynamic intracanal fluid agitation (7-8). The EndoVac System (EV) (Discus Dental, Culver City, CA, USA) is an apical negative pressure irrigation device that is designed to drain irrigating solutions at the apical third of canal by overcoming the vapor lock effect (9). The laser helps in smear layer removal by its combined effect of photoablation and photoacoustic streaming (10). Er: YAG laser has been proven to be the most efficient among the available laser systems in smear layer removal and has also been approved by FDA to be used in endodontics (11-12).

This study was conducted for an *in vitro* comparative evaluation of the smear layer removing efficacy of these three systems (EA, EV, and Er:YAG laser) at the apical, middle and coronal third of root canal under the scanning electron microscope.

## Material and Methods

Recently extracted human mature permanent mandibular premolars were collected from the Department of Oral and Maxillofacial Surgery, King George’s Medical University, Lucknow. The teeth were digitally radiographed using both buccal and proximal views to confirm a single patent root canal devoid of any complex root canal anatomy. Teeth selected had root curvature not greater than 10 degrees and root length not shorter than 12mm.Teeth were then examined under a ×20 magnification laboratory microscope (Stemi DV4 Spot; Carl Zeiss, Oberkochen, Germany) for the absence of any caries, restorations or cracks. Calculus and other soft tissue debris were removed and teeth were autoclaved for 40 minutes to prevent and reduce any microbial growth during storage. The samples were stored in an aqueous solution containing 0.2% thymol to avoid dehydration. Teeth were decoronated, and root length was standardized to 12mm by using a diamond disc operated at low speed.

-Sample preparation:

An ISO size #10 K file (Dentsply Maillefer, Ballagues, Switzerland) was inserted into the root canal until just visible at the apical foramen. The working length (WL) was established 1 mm short of the length. Each apex was sealed with sticky wax to simulate the clinical situation. A coronal reservoir was created for irrigant placement with a size 4 Gates Glidden drill placed 4 mm into the canal (13). The root canals were prepared with ProTaper rotary instruments (Dentsply Maillefer, Ballaigues, Switzerland) up to apical size #40 (F4). The canals were irrigated with 5 ml, 5.25% NaOCl between each file using a 30 gauge needle (NaviTip, Ultradent South Jordan, UT) placed 1mm from the WL. The apical patency was checked after each instrument with a #10 K-file. At the end of instrumentation, irrigation was done with 3ml saline to remove any remaining NaOCl. The specimens were then randomly divided into four groups according to the activation modality of irrigants used (n=10). In each group the final irrigants used were 10ml, 17% EDTA and 10ml, 5.25% NaOCl, activated according to the manufacturer’s protocol.

-Final irrigation protocols:

GROUP-I: Control (n=10)

10 ml, 17% EDTA was delivered using a 30 gauge side vented needle (NaviTip) and left in place for 1 minute per canal. The procedure was then repeated with 10 ml, 5.25% sodium hypochlorite.

GROUP-II: EndoActivator /Sonic activation group: (n=10)

Each canal was irrigated with 10 ml, 17% EDTA using 30 gauge needle (NaviTip). The red (25/04) EndoActivator tip was used to activate intracanal solution at a speed of 10 kHz for 1minute (14). The procedure was repeated with 10 ml,5.25% sodium hypochlorite for 1 minute. The protocol used was as suggested by Ruddle (14).

GROUP-III: EndoVac/Apical negative pressure group: (N=10) 

30 seconds period of irrigation with 2.5ml, 5.25% NaOCl was done by using the master delivery tip while the macrocannula was constantly moved up and down in the canal. This was followed by leaving the canal full of irrigant for 30s. Three irrigation cycles using the microcannula placed at full working length followed. The first cycle was 30 s of 2.5 ml,5.25% NaOCl followed by 30 s of soaking; the second cycle was 1 min of 10ml, 17% EDTA followed by 1 min of soaking; and the third cycle was 1 min of 5ml, 5.25% NaOCl followed by 1 min of soaking. The protocol is similar to that used by Parente (15).

GROUP-IV: Er:YAG laser: (N=10)

 Er: YAG laser (2940 nm) with R-14 handpiece and 300-µm endodontic fiber tip (AT Fidelis; Fotona, Ljubljana, Slovenia) was used at 50 mJ repetition rate of 10Hz at 0.5 W (13) without air- water supply. 10 ml of 17% EDTA was deposited with 30 gauge needle (NaviTip) into the canal. The laser tip was kept stationary at 5mm from the working length and activated for three cycles of 15 seconds each with resting time of 5 seconds. The procedure was then repeated with 10ml, 5.25% NaOCl. The protocol is similar to that used by Ross (16).

Sample Preparation:

The roots were grooved longitudinally on the external surface with a diamond disc without penetration into the root canals and then split into two halves with a chisel. For each root, the half containing the most visible part of the endodontic wall was conserved (17). Selected half was divided into three sections by making grooves at 4 and 8 mm from the root apices by using a diamond bur. This was done to define the coronal, middle, and apical thirds. Each section was then secured on metal stubs, desiccated, sputter-coated with gold, and viewed with scanning electron microscopy.

**Figure 1:**
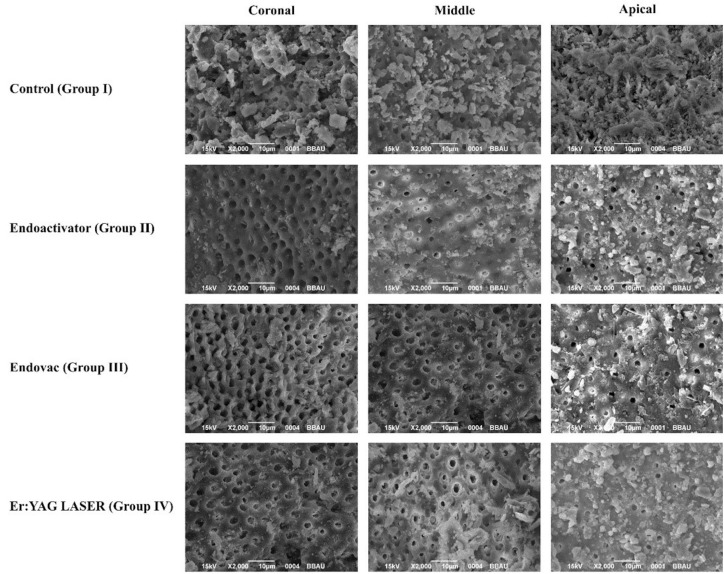
SEM photomicrographs of the four groups at coronal, middle and apical third.

-Scanning electronic microscope evaluation:

Smear layer removal was evaluated by the photomicrographs taken at 2,000 magnifications (Fig. 1). Four observers performed blind evaluation independently after examining the photomicrographs. The assessment was repeated by each observer after 15 days.

A 5-score index system codified by Hulsmann *et al.*, (18) which measured the presence, quantity, and distribution of the smear layer was used to measure the smear layer removing efficiency. Score1 = no smear layer (dentinal tubules open), score 2= small amount of smear layer (some dentinal tubules open), score 3 = homogenous smear layer covering the root canal wall (only a few dentinal tubules open), score 4 =complete root canal wall covered by a homogenous smear layer (no open dentinal tubules), score 5 =heavy non-homogenous smear layer covering the complete root canal wall.

The Kappa test verified intraexaminer and interexaminer reliability for scanning electron microscopic assessment. The differences between irrigation techniques were compared non-parametrically using Kruskal-Wallis and Mann-Whitney U tests, *P* values were computed and compared with statistical significance at the *P*=0.05 level. All statistical analyses were performed using IBM SPSS 20 software (IBM SPSS Inc., Chicago, IL).

## Results

**Figure 2:**
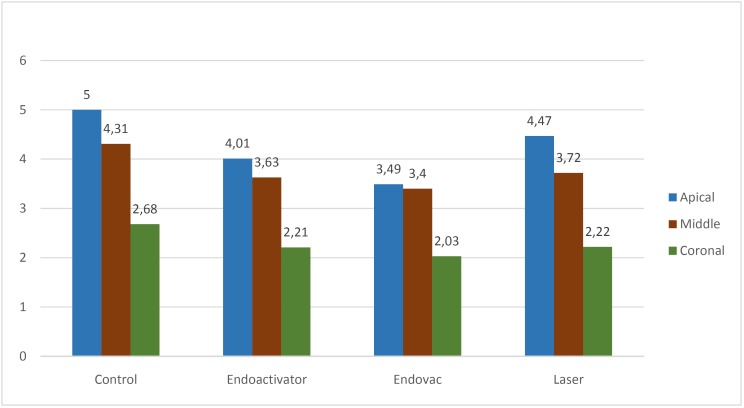
Comparison of mean score among different groups at coronal, middle and apical third.

Kappa test results showed a strong intra and inter-examiner agreement at both Day 1 and 15 ( Table 1, Table 2). The scores at coronal, middle and apical third for all four groups was calculated as the mean score and standard deviation ( Table 3, Fig. 2). At the apical third the mean score was highest for Control (5.0) followed by laser (4.47) and EA (4.01) and least for EV (3.49). The P value was significantly different when Control was compared with EV (*P*=0.0001), EA (*P*=0.01) and laser (*P*=0.033). At the apical third, the cleaning efficacy of EV was better when compared to EA (*P*= 0.039) and laser (*P*=0.0001). EA proved to be better than laser in cleaning the smear layer at the apical third (*P*= 0.04). At the middle third mean score was highest for Control (4.31), followed by EA (3.63) and laser (3.72) and least for EV (3.40). There was a significant difference when Control was compared with EA (*P* = 0.0001), EV (*P*= 0.0001) and laser (*P*=0.00014). However, there was no significant difference seen between EA, EV, and laser (*P* >0.05). At the coronal third the mean score for Control (2.68) was highest, there was not much difference between the mean scores of EA (2.21), EV (2.03) and laser (2.22). However, there was no significant difference between the four groups (*P*>0.05).

**Table 1:**
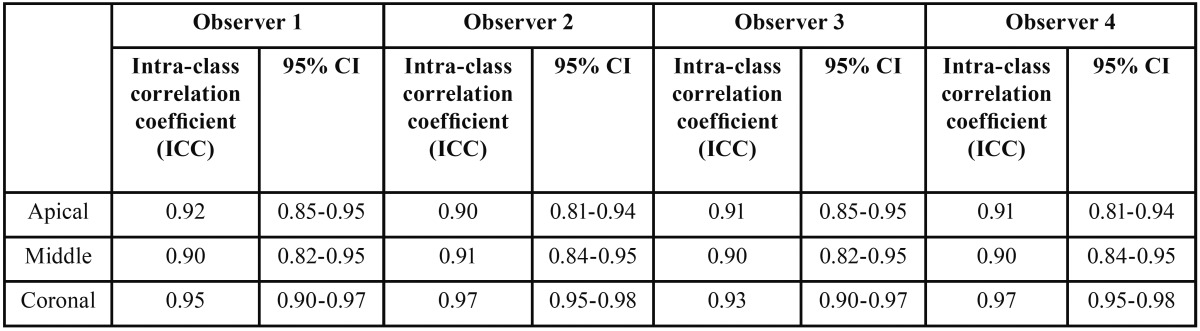
Comparison of inter-observer agreement at Day 1 and Day 15 at apical, middle and coronal third.

**Table 2:**
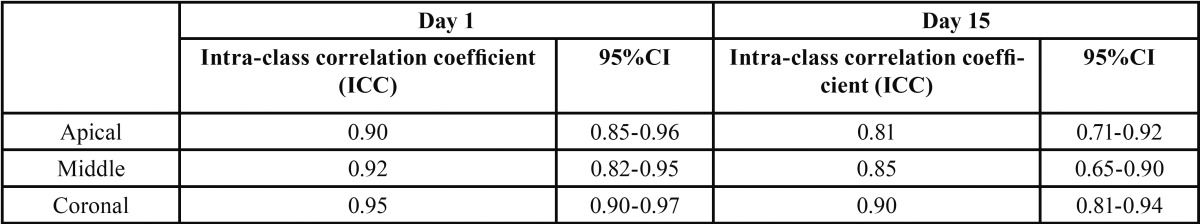
Comparison of intra-observer agreement from Day 1 to Day 15 at apical, middle and coronal third.

**Table 3:**
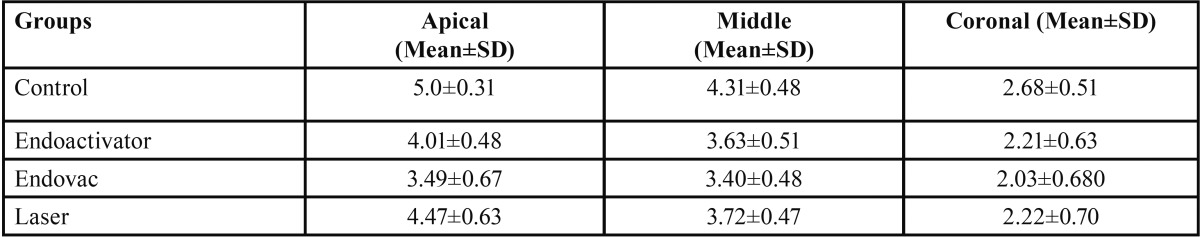
Comparison of mean score among different groups at apical, middle and coronal Third.

## Discussion

The aim of this study was to evaluate the effect of three different irrigating systems (EndoVac, EndoActivator, and Er:YAG laser) in removing the smear layer at the apical, middle and coronal third of the dentinal wall. Past studies have compared these three systems individually with needle irrigation (19-21). Few studies have also compared EA with EV (22) or laser (23), but till now no study has attempted to compare the smear layer removing efficacy of EndoVac, EndoActivator, and Er: YAG laser (with a plain fiber tip) with a constant volume of irrigant.

Bias can occur while selecting the images by SEM operators and also while scoring the SEM images by examiners (18). In this study, Kappa values showed excellent intraexaminer and interexaminer concordance at two separate time periods.

Although an *in vivo* scenario is preferred, the advantages of an *in vitro* study are the ability to ensure uniformity and control of variables. In this study, the apex of teeth was sealed with wax to simulate *in vivo* conditions such as gas entrapment in the root canal and periodontal ligament (22). A well-shaped and fully tapered canal is necessary to act as an adequate reservoir of irrigant (24). Instrumentation to size #40 is required for an efficient irrigation for both positive and negative pressure systems (25). Hence, Gates Glidden drill #4 (Dentsply, USA) was used to create a coronal reservoir for the irrigant, and biomechanical preparation was completed with Protaper rotary files F4. Between each instrumentation, canals were irrigated with 5.25% NaOCl because of its antimicrobial and tissue dissolving properties (26). For the final irrigation the recommended combination of 10 mL,17% EDTA and 10 ml,5.25% NaOCl was used (27-29).

In the present study, selected laser was Er:YAG laser. The mid-infrared erbium lasers are highly absorbed in water and hydroxya-patite in comparison to visible and near-infrared electromagnetic radiation and hence are more efficient for smear layer removal and disruption of intracanal biofilms (30-31).

Laser irradiation may result in a potentially hazardous effect in periodontal tissue (32). The selected Er:YAG laser has lower thermal effects and hence lower thermal damage to the surrounding dental tissues (33-35). Moreover, 5 seconds resting period between each activation recommended by Gutknechet *et al.* was used to lower the thermal effects (36).

At the apical third, the mean score was highest for Control (5.0). Previous studies have proved needle irrigation in a closed system to be ineffective in delivering adequate volume and pressure of irrigant at the apical third (37-38). With a conventional syringe irrigation, the irrigating solution is delivered only 1 mm deeper than the tip of the needle (39). This limits the penetration depth of the irrigating solution resulting in less effective smear removal from the apical third (40-41).

The apex of the samples in this study was sealed with glue and thus behaved as a closed-end channel. This might have resulted in gas entrapment at its closed end, producing the vapor lock effect (15). Except for EV, this phenomenon might have been present in all three groups (i.e. EA, laser and needle irrigation). In samples irrigated with EV, due to a continuous supply of fresh irrigant being delivered by negative pressure, vapor lock effect might have been avoided, resulting in better cleaning in the apical third (19).

At the apical third, the cleaning efficacy of EV was significantly better than needle irrigation. A similar result was described by Heilborn *et al.*; (42), Parente *et al.*; (15), S. Chris (19) who showed significantly better cleaning with EV compared with traditional positive-pressure irrigation. The apical negative pressure irrigation in EV results in a significantly more volume of irrigant delivered at apical third, without the risk of periapical extrusion (43-44).

EV performed significantly better than EA at apical third. These results are similar to showed by M. Manuele *et al.*; (22). Endo-Activator works on the principle of hydrodynamic agitation of irrigant but acoustic microstreaming can only occur in a liquid phase. Therefore, once a sonic activated tip leaves the irrigant and enters the apical vapor lock, acoustic microstreaming, and cavitation becomes physically impossible (20). Since EA in spite of its hydrodynamic activation cannot overcome the vapor lock effect, it resulted in a less effective cleaning (22). Conversely in a recent study smear layer removing efficacy of EA was found to be better than EV (45).

EA performed better than Needle irrigation at apical third. These results are in contrast to past studies in which no significant difference was reported in smear layer removing efficacy of EV and needle irrigation (46-47). This difference might be due to the lower volume of final irrigant used compared to the present study.

Er:YAG performed better than Needle irrigation at apical third. Similar results have been reported in the past by G. Rebecca (21) and de Groot (48). Laser results in an impulsive activation of irrigant at every pulse in contrast with steady streaming of irrigant with needle activation resulting in more efficient smear removal (21).

In this study, both EV and EA performed better than Er:YAG laser at apical third. This might be attributed to the hydrodynamic movement present with EV and EA resulting in vigorous intracanal fluid agitation (16). Whereas the effect with laser is mainly linear and the optical fiber may not have reached all the surfaces of the root canal walls (48). The difference might also be attributed to the placement of tip. While the tip of both EV and EA was placed at apex, the laser tip was kept stationary at 5mm from the WL.

At the middle third, all the Groups performed better than Control. These results differ from those reported by Nielson and Baumgartner in which EV performed better than needle irrigation at 1mm from WL, but there was no difference seen at 3mm (43). This may be attributed to not keeping the volume of irrigant constant in compared groups, (unlike the present study).

There was no significant difference seen between EA, EV, and laser at the middle third. Similar results were reported by Manuele Mancini *et al.* in which no significant difference was seen in the cleaning efficacy of EV and EA at 3, 5 and 8mm from the apex (22).

 At the coronal third, no significant difference was observed between the four groups. The process of smear layer removal was more efficient in the coronal and middle third than in the apical third of the canal, for all four groups. This is in agreement with previous studies (21,42). The diameter of a root canal decreases on moving from coronal to apical third. Hence while irrigating, the coronal dentin is exposed to a higher volume of irrigants and allows for a better flow of the solutions as compared to apical dentin, resulting in better smear layer removal from coronal third (22).

Recently Er:YAG laser with a conical fiber tip (Photon induced photo acoustic streaming, PIPS) has been introduced that claims to be more effective than Er:YAG laser with a plain fiber tip (49). Conversely, few recent studies proved Er:YAG laser with plain fiber tip to be more efficient in smear removal than that with conical fiber tip (23,50). It was speculated that in PIPS technique since the laser tip is placed at the coronal third, it results in a less effective irrigant activation at apical third of canal. Further studies comparing EA, EV, Er:YAG (PIPS, conical fiber tip) and Er: YAG (plain fiber tip) should be carried out.

## Conclusions

This study concluded that use of EndoActivator, EndoVac, and Er: YAG laser increases the smear layer removing efficiency at apical and middle third. EndoVac was more efficient than other techniques at apical third. EndoActivator performed better than Er: YAG laser at apical third.
